# Impacts of F18^+^ *Escherichia coli* on Intestinal Health of Nursery Pigs and Dietary Interventions

**DOI:** 10.3390/ani13172791

**Published:** 2023-09-02

**Authors:** Marcos Elias Duarte, Yesid Garavito-Duarte, Sung Woo Kim

**Affiliations:** Department of Animal Science, North Carolina State University, Raleigh, NC 27695, USA; mduarte@ncsu.edu (M.E.D.); yrgaravi@ncsu.edu (Y.G.-D.)

**Keywords:** F18^+^ *Escherichia coli*, dietary intervention, intestinal health, nursery pigs, post-weaning diarrhea

## Abstract

**Simple Summary:**

The modern intensive practices in pig production to enhance productivity have increased post-weaning diarrhea (PWD), often caused by F18^+^ *Escherichia coli*. Antibiotics have been widely used in animal production to improve growth; however, their overuse has led to antibiotic-resistant bacterial pathogens, posing risks to both the sustainability of pig production and public health. The pathogenesis of F18^+^ *E. coli* damages the intestinal morphology, barrier function, microbiota composition, and immune responses in nursery pigs, leading to reduced growth performance. Various nutritional interventions have been investigated to combat the PWD issue, including low crude protein formulation, antibacterial minerals, probiotics, prebiotics, postbiotics, and phytobiotics, aiming to enhance intestinal microbial composition, health, and growth performance. Implementing effective strategies to control F18^+^ *E. coli* is crucial for pig production. Therefore, this review is to explore the impact of F18^+^ *Escherichia coli* on pig production, particularly its association with post-weaning diarrhea (PWD) in nursery pigs, and the nutritional strategies to counter its harmful effects.

**Abstract:**

This review focused on the impact of F18^+^ *E. coli* on pig production and explored nutritional interventions to mitigate its deleterious effects. F18^+^ *E. coli* is a primary cause of PWD in nursery pigs, resulting in substantial economic losses through diminished feed efficiency, morbidity, and mortality. In summary, the F18^+^ *E. coli* induces intestinal inflammation with elevated IL6 (60%), IL8 (43%), and TNF-α (28%), disrupting the microbiota and resulting in 14% villus height reduction. Besides the mortality, the compromised intestinal health results in a 20% G:F decrease and a 10% ADFI reduction, ultimately culminating in a 28% ADG decrease. Among nutritional interventions to counter F18^+^ *E. coli* impacts, zinc glycinate lowered TNF-α (26%) and protein carbonyl (45%) in jejunal mucosa, resulting in a 39% ADG increase. Lactic acid bacteria reduced TNF-α (36%), increasing 51% ADG, whereas *Bacillus* spp. reduced IL6 (27%), increasing BW (12%). *Lactobacillus* postbiotic increased BW (14%) and the diversity of beneficial bacteria. Phytobiotics reduced TNF-α (23%) and IL6 (21%), enhancing feed efficiency (37%). Additional interventions, including low crude protein formulation, antibacterial minerals, prebiotics, and organic acids, can be effectively used to combat F18^+^ *E. coli* infection. These findings collectively underscore a range of effective strategies for managing the challenges posed by F18^+^ *E. coli* in pig production.

## 1. Introduction

As pig production adopts more intensive practices to increase productivity and profitability, there is an increase in husbandry-related stressors. These stressors include a wide range of physical, environmental, psychological, and nutritional challenges that increase susceptibility to infectious diseases [1,2,3]. In recent years, there has been an increased incidence of post-weaning diarrhea (PWD) in nursery pigs. Enterotoxigenic *Escherichia coli* (ETEC) is the leading cause of PWD and edema disease (ED) in nursery pigs [4,5,6]. Post-weaning diarrhea typically appears between the second and third week after weaning, causing watery diarrhea that can range in color from yellowish gray to slightly pink and can last up to 10 d [7,8,9,10]. In general, the mortality could be around 1.5 to 2% in post-weaned and/or grow-finish pigs with moderate diarrhea and up to 25% in untreated pigs with severe to moderate diarrhea [11]. In addition to the impacts on animal health, the ETEC represents an economic impact on the swine industry due to morbidity, mortality, decreased weight gain, and the cost of treatment, vaccinations, and feed supplements [10]. The control of ETEC infection has represented a challenge to researchers and producers around the world [12,13,14].

Generally, ETEC utilizes specific fimbriae or pili to establish colonization by adhering to the enterocytes in the small intestine [15,16,17]. The type of fimbriae necessary for colonization depends on the age of the pig [18]. Nursery pigs with PWD are typically associated with the F4 and F18 fimbriae [19,20]. F18^+^ *E. coli* has been identified as a prevalent strain in nursery pigs with PWD worldwide and is a significant health concern for their growth and intestinal health. The prevalence rates, ranging from 12% to 44% in different regions of the world, emphasize the need for ongoing research and control measures [5,21,22,23]. The findings highlight the global impact of ETEC as a major health concern for nursery pigs, particularly in its association with decreased growth performance and intestinal health issues.

Because of the spread of F18^+^ *E. coli* in pig farms around the world, various strategies have been used to control its spread, one of which has been the use of antibiotics, which has contributed to an increase in antibiotic-resistant bacterial pathogens in pigs and humans [24]. As an example, Pedersen et al. [25] observed that an outbreak of F18^+^ *E. coli* persists in nursery pigs despite antibiotic treatment in 58% of the herds studied. In order to control the spread of F18^+^ *E. coli* in weaned pigs, different alternatives must be used, considering the previously mentioned affected factors of pig health, mortality, economic losses in pig production, and public health. Therefore, multiple investigations on the problem of F18^+^ *E. coli* in pig production, specifically its impact on the intestinal health of nursery pigs and the dietary interventions used to ameliorate its deleterious effects, have been assembled in this review article.

## 2. Virulence Factors of F18^+^ *Escherichia coli*

**Figure 1:**
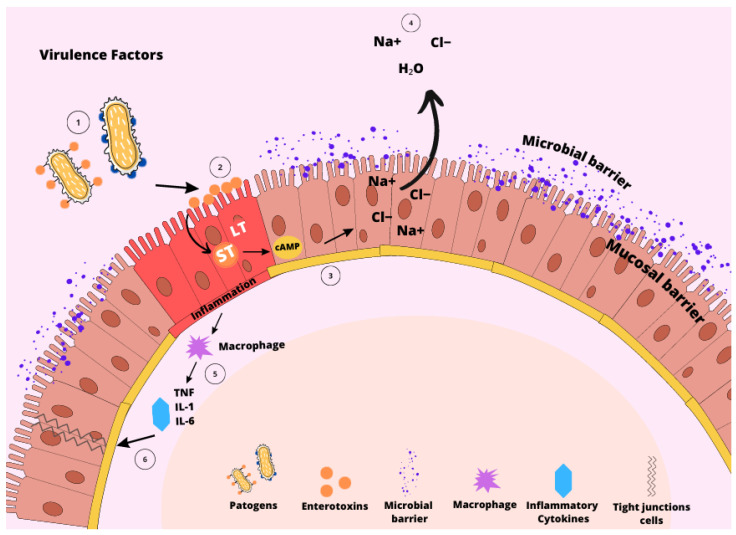
Virulence factors of F18^+^ *Escherichia coli*. The colonization by F18^+^ *E. coli* starts with adhering to the intestinal epithelial cells (1), facilitated by one or more virulence factors, like fimbriae F18, and diffuse adherence (AIDA). The attachment, in addition to the presence of lipopolysaccharide (LPS), initiates the interaction with the intestinal surface (2) through receptors found in the mucin layer. Subsequently, heat-labile toxin (LT) and heat-stable toxins (ST) enterotoxins are generated and bind to their respective receptors. This binding triggers the production of cellular cyclic adenosine monophosphate (cAMP) (3), setting off a cascade of reactions that ultimately lead to the flood of chloride, sodium, and water ions into the intestinal cell lumen (4). These series of events initiate the inflammatory responses (5) by prompting the epithelial cells to release inflammatory cytokines (6).

The main route of contamination of ETEC, including F18^+^ *E. coli*, is by ingestion, then it passes through the stomach until it reaches the small intestine. When ETEC adheres to the epithelium of the small intestine, colonization occurs, followed by rapid proliferation and production of one or more types of enterotoxins [26,27,28,29]. The major virulence factors of F18^+^ *E. coli* include adhesins, including fimbriae F18, and adhesin involved in diffuse adherence (AIDA), lipopolysaccharide (LPS), and enterotoxins [28,29,30,31]. These factors are pathogen-associated molecular patterns (PAMP) that interact with the host through pattern recognition receptors (PRR), consequently triggering inflammatory responses [32] (Figure 1).

### 2.1. Fimbriae

One of the virulence characteristics of ETEC is to adhere to the epithelium of the small intestine without causing significant morphological changes. Fimbriae are structural proteins that form the backbone filament on certain bacterial cells and are classified into several subtypes based on antigenic differences. There are two known variants of F18 fimbriae. The F18ab is linked to ED-causing strains, whereas F18ac is linked to PWD-causing strains; both variants were previously known as F107 and 2134P or 8813, respectively [20,33]. The attachment of F18 fimbriae with specific receptors promotes F18^+^ *E. coli* colonization in the small intestine [34]. Coddens et al. [35] have identified glycolipids having blood group HBGAs as receptors for F18 fimbria in the small intestine of pigs. According to Nagy et al. [36], the presence and function of these adhesion receptors have a significant impact on pig’s susceptibility to F18^+^ *E. coli* infections. After adhesion, fimbriae perform a variety of other functions in the intestine that include interacting with immune cells, promoting biofilm formation, promoting intestinal persistence, and facilitating bacterial aggregation [37]. Furthermore, Toll-like receptor 5 (TLR5) expressed in the small intestine has been reported to play a role in the increased expression of pro-inflammatory cytokines in weaned pigs [38].

### 2.2. Adhesin Involved in Diffuse Adherence

It is well known that expressing fimbriae by *E. coli* is the most important mechanism utilized to adhere in the small intestine of nursery pigs. However, there is evidence of a non-fimbriae adhesin identified as adhesin involved in diffuse adhesion (AIDA), which has been associated with ETEC strains in nursery pigs with PWD [31,39,40]. The expression of different adherence systems by *E. coli* can be one of the reasons for the increased incidence of PWD in pigs. According to Niewerth et al. [41], AIDA is also associated with PWD and ED caused by F18^+^ *E. coli* in pigs. Studies have demonstrated that AIDA exerts a significant impact on the immune response by binding with the complement decay-accelerating factor (CD55), thereby activating the phosphatidylinositol 3-kinase (PI3K) and subsequently promoting the expression of the major histocompatibility complex (MHC) class I-related molecule (MICA) on the cell surface [42] and the recruitment and activation of innate immune cells through cytokine and chemokine signaling at sites of inflammation [43].

### 2.3. Lipopolysaccharides

*Escherichia coli* is classified as Gram-negative, a group of bacteria that contains LPS on their cell outer layer. Lipopolysaccharide is known as a potent inflammatory stimulator [44]. In the intestine, the presence of LPS is detected by the Toll-like receptor 4 (TLR4), a PRR expressed in different cells in the body, including the enterocytes, triggering a robust cascade of cytokine responses, potentially culminating in septic shock and death [45,46]. According to Guo et al. [47], LPS can downregulate the expression of tight junction proteins, increasing intestinal permeability and mucosal damage. The authors linked these results to the TLR4/FAK/MyD88 signal transduction axis. However, it is important to mention that commensal bacteria in the gastrointestinal tract are composed of both Gram-negative and Gram-positive bacteria that, in healthy status, interact with the immune system in a balanced manner [8,32]. *Escherichia coli* infection can disrupt this balance, increasing the abundance of Gram-negative bacteria, increasing inflammation and oxidative damage, and disrupting the barrier function [7,8,32]. 

### 2.4. Enterotoxins

Furthermore, the ETEC secrete enterotoxins, which are proteins or peptides that disrupt the normal functions of enterocytes, resulting in increased secretions and decreased absorption [36]. Enterotoxin secretion by *E. coli* is the primary cause of diarrhea by ETEC infection [48]. The *E. coli* enterotoxins are classified based on their thermal stability, high-molecular-weight heat-labile toxin (LT), and low-molecular-weight heat-stable toxins (ST), enteroaggregative heat-stable enterotoxin (EAST-1) and Shiga toxin (Stx) [49,50]. In general, the F18^+^ *E. coli* strain frequently produces STa and STb, whereas LT, EAST-1, and STx are encountered with lower frequency in isolates from pigs with diarrhea caused by F18^+^ *E. coli* [50]. However, STa enterotoxin is more frequently related to neonatal diarrhea and STb with PWD [51]. The genes that encode these peptides are estA and estB, which are found in plasmids [52].

#### 2.4.1. STa

Currently, two subtypes of STa are known. STaP (19 amino acids) and STaH (18 amino acids, were initially isolated from porcine and human ETEC strains, respectively. STaP can be found in porcine, bovine, and human ETEC strains, while STaH can only be found in human ETEC strains [53,54]. The STa enterotoxin binds to guanyl cyclase C (GC-C), a membrane-spanning protein with an extracellular binding domain as well as intracellular protein kinase and catalytic domains. When STa binds to GC-C, it activates guanylate cyclase and raises cyclic guanosine monophosphate (cGMP) levels in enterocytes, which is a glycoprotein at the brush border membrane. This union takes place because STa is a structural analog of the hormone guanylin; this hormone appears to play a role in the regulation of fluid and electrolyte absorption in the intestine [18,55]. In general, STa’s toxic activity is caused by the activation of an intracellular signaling cascade; STa increases the cellular accumulation of cGMP when it binds to guanylate cyclase-C on the apical side of enterocytes. Accumulation of cGMP activates cGMP-dependent protein kinase II (PKII), resulting in phosphorylation of the cystic fibrosis transmembrane regulator (CFTR) and chloride and carbonate ion secretion [23], which results in watery diarrhea, increased secretion of water and electrolytes (Na^+^ and Cl^−^), decreased fluid absorption and causing dehydration and acidosis [47,56]. Furthermore, STa has been linked to moderate intestinal barrier dysfunction, which affects trans-epithelial resistance (TER) [57].

#### 2.4.2. STb

The STb enterotoxin is a peptide of 48 amino acids with four cysteine residues involved in disulfide bridge formation [50]. It is thermostable but susceptible to degradation by proteolytic enzymes. STb is mostly associated with porcine ETEC; however, it has occasionally been detected in ETEC of human origin [18]. Rousset et al. [58] and Chao and Dreyfus [59] reported that STb adheres to microvilli in the jejunum portion, stably associating with the membrane lipid bilayer, which may allow STb bound to the lipid bilayer to cross and be released into the membrane. The authors demonstrated that STb has a specific affinity for sulfatide on the surface of intestinal epithelial cells in the jejunum and constitutes the first step in inducing secretory diarrhea in the intestinal lumen of animals [60,61]. The effect of STb binding to the receptor induces Ca^2+^ uptake in the cells, resulting in the duodenal and jejunal secretion of water and electrolytes. Additionally, STb intoxication causes a significant accumulation of Na^+^ and Cl^−^ at the intraluminal level, which stimulates the secretion of bicarbonate (HCO_3_^−^) [50,62]. The enterotoxin STb also stimulates arachidonic acid metabolism in epithelial cells, which results in elevated PGE2 levels and induces diarrhea [63]. The STb enterotoxin causes tight junction complexes to open, as evidenced by a significant decrease in TER and an increase in paracellular permeability [63,64]. The mechanisms described for ST affecting tight junctions occur because of a decrease in the tight junction proteins zonula occludens and occludin, as well as high levels of intracellular Ca^2+^ in response to STb altering claudin-a protein, which is important for tight junction integrity [52].

## 3. Pathogenesis of F18^+^ *Escherichia coli*

As mentioned before, ETEC infection occurs orally and then colonizes the small intestine by binding to receptors on the small intestinal epithelium or within the mucus layer that covers the epithelium [56]. The fimbriae adhere to specific receptors on the cell membrane of intestinal epithelial cells, as well as to specific or nonspecific receptors in the mucus that regenerate the epithelium [18]. The susceptibility to F18^+^ *E. coli* infections is mainly dependent on the activity of the FUT1 gene, which encodes alpha (1,2)-fructosyltransferase [10]. Pigs with at least one copy of the receptor’s dominant allele are susceptible to colonization [10]. However, it can be speculated that other adhesion mechanisms, including AIDA, can help F18^+^ *E. coli* colonization, even in pigs with resistance to F18 fimbriae. 

**Figure 2:**
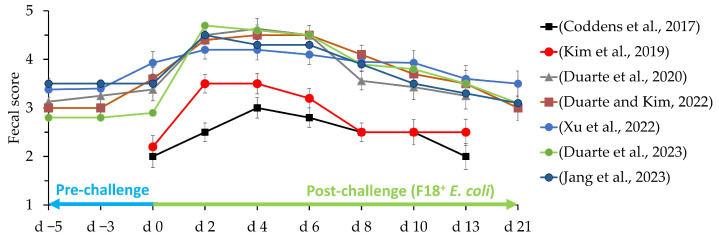
Fecal scores of pigs challenged with F18^+^ *E. coli*. Fecal scores: 1: very hard and dry stool, 2: firm stool, 3: normal stool, 4: loose stool, and 5: watery stool with no shape. Adapted from Coddens et al. [65] (10^11^ CFU), Kim et al. [66] (3 × 10^10^ CFU), Duarte et al. [67] (6 × 10^9^ CFU), Duarte and Kim [8] (4.6 × 10^9^ CFU), Xu et al. [7] (2.4 × 10^10^ CFU), Duarte and Kim [16] (5.2 × 10^9^ CFU), Jang et al. [68] (1.2 × 10^10^ CFU).

Pigs with PWD are typically depressed, with a reduced appetite and a rough, sticky, wet hair coat. Sudden deaths can occur, especially at the beginning of an outbreak, and dead pigs are typically dehydrated with sunken eyes [13]. Some of the symptoms that can be observed by ETEC at the intestinal level are dilation of the small intestine, slight edema, and hyperemic effects. The stomach is slightly distended and filled with dry food, with fundal hyperemia. Lymph nodes in the mesenteric region are enlarged and often appear hyperemic [10]. According to Luppi [13], the PWD caused by *E. coli* generally occurs within 2 to 3 weeks post-weaning and, in some cases, within 6 to 8 weeks post-weaning. The symptoms of PWD caused by F18^+^ *E. coli*, often reported as increased fecal score, can persist for up to 10 days (Figure 2) and present a range of colors from yellowish gray to slightly pink [7,8,9,10]. However, according to Duarte and Kim [8], the F18^+^ *E. coli* can modulate the mucosa-associated microbiota in the jejunum up to 21 days after challenge, increasing the inflammatory status in the intestine of pigs.

## 4. Physiological, Clinical, Immunological, and Growth Responses of Pigs

The immunological and physiological responses of pigs following pathogenic infections may be altered due to the physiological state of the animal [69]. In pigs, the microbial colonization process begins early in life and is induced by the maternal microbiota and maternal immunity. This process is critical in host-autonomous microbial mutualism [70]. Pig microbiota has a high population density and a wide and complex diversity of interactions through the intestinal tract [32,71]. It has been observed that the diversity of *E. coli* phenotypes in pigs increases as the pigs grow, which can be attributed to changes in the physicochemical characteristics of the intestinal tract [72]. Moredo et al. [73] observed that ETEC presence found in lactating pigs was 16%, whereas the population found in nursery pigs was 66%.

The mucosal layer serves as a crucial defense mechanism against the invasion of microorganisms into the intestinal tract [8,32]. This specialized layer lines the interior surface of the gastrointestinal tract, providing lubrication to the luminal contents and acting as a robust physical barrier, preventing the entry of bacteria and other antigenic substances; the structural component of the mucus layer is mucin, which is secreted by goblet cells [74,75]. It has been observed that mucus protein mucin 2 (MUC2) gene expression increases in response to F18^+^ *E. coli* infection and is more prolonged during the peak of infection, demonstrating the role of mucin as the first line of defense against infection due to F18^+^ *E. coli* [63]. 

An important role of F18^+^ *E. coli* infection is disrupting the balance of the intestinal microbiota composition, affecting the immune system even after pigs recover from clinical symptoms that last for 7 to 11 days [8,16,67,68,76,77,78,79,80,81]. According to Duarte and Kim [8], the mucosa-associated microbiota showed changes due to *E. coli* infection 21 days after the challenge. Bacteria belonging to Proteobacteria, mainly *Helicobacteraceae, Campylobacteraceae, Pseudomonadaceae,* and *Enterobacteriaceae*, showed increased abundance in the intestinal mucosa of pigs challenged with F18^+^ *E. coli* [7,8,16,67]. The disrupted balance of the microbiota composition in jejunal mucosa caused by F18^+^ *E. coli* infection has been correlated with increased inflammation in the intestines of pigs [8]. Infection with F18^+^ *E. coli* has been shown to affect specific systemic and local inflammatory responses in the small intestine, increasing the population of white blood cells, pro-inflammatory cytokines, particularly TNF-α in serum, and the number of neutrophils and macrophages in the ileum [8,66]. According to Loos et al. [82], there seems to be a general antibacterial response, which expresses innate immunity genes in the intestinal mucosa, including PAP, MMP1, and IL8, as well as a specific response based on enterotoxin. In the case of ST, the response is mediated by genes such as IL17A and IL1B. Studies have demonstrated high concentrations of pro-inflammatory cytokines in pigs up to 21 days after oral inoculation with F18^+^ *E. coli* [8,16,76,77,78,79,80,81]. According to previous studies, pigs with F18^+^ *E. coli* infection have increased concentrations of IL6 (60%) [67,77], IL8 (43%) [7,67,83], and TNF-α (28%) [7,8,16,67,68,74] in the small intestine of nursery pigs. The increased inflammatory response in the intestinal mucosa leads to increased products of oxidative stress and damage to the intestinal epithelium [8,84]. According to McLamb et al. [69], the intestinal epithelium was affected when pigs were exposed to F18^+^ *E. coli*. The authors reported a decrease in villus height and a noticeable change in morphological appearance in pigs between 16 and 20 days after weaning. 

**Figure 3:**
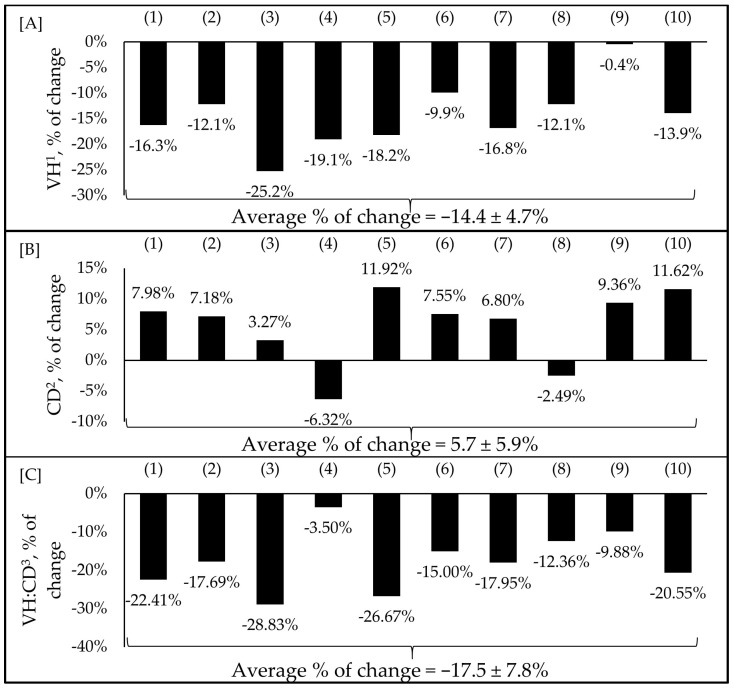
Impacts of F18^+^ *E. coli* challenge on villus height (**A**), crypt depth (**B**), and villus height to crypt depth ratio (**C**) of nursery pigs. Selected studies employed F18^+^ *E. coli* as the challenge model, encompassing both negative and positive control treatments and reported outcomes related to intestinal morphology. The selected studies were (1) Xu et al. [7], (2) Duarte and Kim [8], (3) Duarte et al. [16], (4) Kim et al. [66], (5) Duarte et al. [67], (6) Jang et al. [68], (7) Sun et al. [76], (8) Li et al. [79], (9) Liu et al. [81], (10) Chang et al. [85]. The percentage of change refers to statistically significant (*p* < 0.05) and tendency (0.05 ≤ *p* < 0.10) effects of F18^+^ *E. coli* compared with the negative control on the intestinal morphology reported from each respective study. ^1^ Villus height. ^2^ Crypt depth. ^3^ Villus height to crypt depth ratio.

In recent years, F18^+^ *E. coli* infection has become more frequent [11]. Studies using the F18^+^ *E. coli* challenge model are necessary to understand the consequences and the alternatives to mitigate this infection [7,8,16,66,67,68,77,79,81,85]. Studies using the F18^+^ *E. coli* model have shown a 14% reduction in villus height ranging from −0.4 to −25%, a 6% increase in crypt depth ranging from −6.32 to 11.92%, and a reduction in villus height to crypt depth ratio (VH:CD) ranging from −3.5 to −28.8% (Figure 3A–C) [7,8,16,66,67,68,76,79,81,85]. 

The reduced villus height and increased cell proliferation in the crypts as a consequence of F18^+^ *E. coli* were previously correlated with Proteobacteria, including *Helicobacter* spp. [7,8]. Crypt depth and the VH:CD ratio are utilized as markers for assessing enterocyte proliferation and villus damage [8]. Selected studies employed F18^+^ *E. coli* as the challenge model, encompassing both negative and positive control treatments and reported outcomes related to intestinal morphology. The percentage of change refers to statistically significant (*p* < 0.05) and tendency (0.05 ≤ *p* < 0.10) effects of F18^+^ *E. coli* compared with the negative control on the intestinal morphology reported from each respective study. There were no correlations between the dose of F18^+^ *E. coli* and the variables of intestinal morphology. Therefore, the average percentage of change reported in Figure 3 is regardless of the dose of the inoculum within each study. 

Exposure to F18^+^ *E. coli* has also been found to affect both transcellular and paracellular permeability in the jejunum of nursery pigs [66]. The impact of altered intestinal morphology, including reduced villus height, can decrease the efficiency of nutrient absorption and utilization, consequently reducing the growth performance of pigs [67]. 

**Figure 4:**
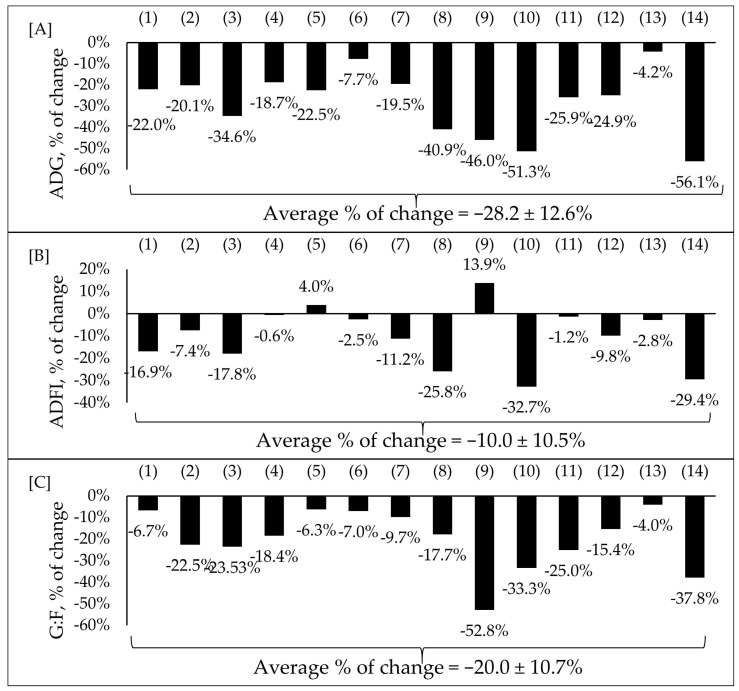
Impacts of F18^+^ *E. coli* challenge on average daily gain (ADG) (**A**), average daily feed intake (ADFI) (**B**), and gain-to-feed ratio (G:F) (**C**) of nursery pigs. Selected studies employed F18^+^ *E. coli* as the challenge model, encompassing both negative and positive control treatments and reported outcomes related to growth performance. The selected studies were (1) Xu et al. [7], (2) Duarte et al. [16], (3) Kim et al. [66], (4) Duarte et al. [67], (5) Jang et al. [68], (6) Sun et al. [76], (7) Wong et al. [77], (8) Li et al. [79], (9) Liu et al. [81], (10) Becker et al. [83], (11) Chang et al. [85], (12) He et al. [86], (13) Jerez-Bogota et al. [87], (14) Caprarulo et al. [88]. The percentage of change refers to statistically significant (*p* < 0.05) and tendency (0.05 ≤ *p* < 0.10) effects of F18^+^ *E. coli* compared with the negative control on the growth performance reported from each respective study.

Multiple research investigations have linked F18^+^ *E. coli* to PWD and reduced growth performance [7,16,66,67,68,76,77,79,81,83,85,86,87,88]. These studies on nursery pigs challenged with F18^+^ *E. coli* showed an average reduction of 27% in ADG, 10% in ADFI, and 20% in feed efficiency (Figure 4A–C). 

Selected studies employed F18^+^ *E. coli* as the challenge model, encompassing both negative and positive control treatments and reported outcomes related to growth performance. The percentage of change refers to statistically significant (*p* < 0.05) and tendency (0.05 ≤ *p* < 0.10) effects of F18^+^ *E. coli* compared with the negative control on growth performance reported from each respective study. There were no correlations between the dose of F18^+^ *E. coli* and the variables of growth performance. Therefore, the average percentage of changes reported in Figure 4A–C is regardless of the dose of the inoculum within each study. These results could be due to the differences in age, initial body weight, genetics, duration of trial, and basal diet composition among studies that could affect the response of pigs to ETEC.

The diminished growth response observed can be attributed to the impact of F18^+^ *E. coli* on intestinal health. The disrupted intestinal health contributes to the development of intestinal malabsorption syndrome, leading to a decline in nutrient absorption and, consequently, reduced feed efficiency [16,77].

## 5. Nutritional Interventions

To prevent or mitigate the severity of F18^+^ *E. coli* infection in nursery pigs and enhance their growth performance, nutritional strategies have emphasized the improvement in feed quality, meeting animal nutritional requirements. The use of feed additives to minimize the negative effects of anti-nutritional factors and modulate the intestinal microbiota has also been used as a strategy to enhance the resiliency of pigs to potential pathogens (Table 1). Extensive studies have been conducted on the influence of dietary interventions in modulating the composition of the intestinal microbiota, establishing a significant link to the promotion of intestinal health [32]. The growth of beneficial bacteria promoted by dietary intervention can further improve intestinal health, reducing the susceptibility and severity of *E. coli* infection [7,8,9,16,32,66,67,68]. However, future research should further explore the interaction of intestinal microbiota with the intestinal mucosa, evaluating the changes in PRR and the functionality of the microbiota in challenged pigs influenced by dietary interventions. 

Global regulations limiting the use of specific additives, such as antibiotics as growth promoters, ZnO, and Cu, have prompted the search for alternative methods to support both animal growth performance and intestinal health. Moreover, the price fluctuation of ingredients has stimulated the use of alternative ingredients and the simplification of nursery diets.

### 5.1. Low Crude Protein Diets

Among the nutritional interventions, low crude protein diets have been long used as a strategy to reduce the incidence of PWD in pigs [90,91,92,93,94,95]. A typical nursery diet is characterized by a higher crude protein and lower fiber content, which can promote the growth of proteolytic bacteria [71]. Excessive amounts of undigested protein can turn the intestinal environment propitious to the proliferation of opportunistic pathogens, including *E. coli*, increasing the chances of infection [93]. The unbalanced microbiota composition can increase intestinal inflammation, disrupting the epithelial barrier and consequently reducing growth performance [8,32]. Low crude protein formulations are effective in reducing PWD in pigs [91]. Although reducing the PWD, some studies showed a reduction of both PWD and growth performance by reducing the crude protein in the diet, even with supplemental amino acids to meet the requirements [90,91,92]. According to Rocha et al. [95], the reduction in crude protein in the diet of pigs can turn deficient some non-essential amino acids that may have functional activities. The authors suggested that the crude protein in the diet of nursery pigs can be reduced by up to 18.4% without compromising the growth performance of pigs. Luise et al. [94] reported that reduced dietary crude protein can decrease PWD, possibly by reducing the pH, protein fermentation, and the expression of genes related to inflammation of the intestinal mucosa, including TLR4, possibly by modulating the microbiota toward a healthier composition.

### 5.2. Zinc

Zinc is a trace element that holds significant importance in nutrition, growth, and immunity. Traditionally, it has been administered in the form of zinc oxide (ZnO) at pharmacological doses up to high doses of 2000 to 3000 mg/kg in diets for weaned pigs. This approach serves as an antibiotic alternative, aiming to prevent intestinal inflammation and enhance weight gain [96,97]. Wang et al. [98] reported that a ZnO dose of 1200 mg/kg in the diet improves intestinal integrity, improves weight gain, and significantly reduces the *E. coli* population in weaned pigs. However, alternatives have been studied to reduce the use of ZnO in pig diets due to restrictions related to environmental pollution and microbial resistance [68,96,99,100,101,102,103]. Kociova et al. [96] used a zinc phosphate-based nanoparticle supplemented to the diet for weaned pigs at 500, 1000, and 2000 mg of Zn per kg of feed and observed a significant increase in pig weight (20%) and antioxidant status and a decrease in the occurrence of diarrhea at 500 mg/kg. Jang et al. [68] reported that supplementation of zinc glycinate at 400 to 675 mg/kg in the diets replacing zinc oxide reduced the deleterious effects of F18^+^ *E. coli* by increasing the abundance of *Enterobacteriaceae,* whereas increasing Actinobacteria in jejunal mucosa, reducing IL8 (39%), TNF-α (26%), MDA (31%), and protein carbonyl (45%) in jejunal mucosa, consequently increasing ADG by 39%, and reducing fecal score of pigs. Additionally, coated or encapsulated ZnO has also been an alternative to improve intestinal health, improving growth performance at lower doses compared to traditional ZnO, consequently reducing environmental excretion [99,100,101,102]. Kim et al. [103] reported that the dietary supplementation of encapsulated ZnO at 100 mg/kg reduced diarrhea, increased growth performance, and goblet cell count in the small intestine of nursery pigs challenged F4^+^ ETEC. These results indicate that encapsulated ZnO at lower doses can also prevent the deleterious effects of other pathogens, including F8^+^ *E. coli.* However, to date, there is no study reporting the efficacy of encapsulated ZnO in preventing specifically F18^+^ *E. coli.*

### 5.3. Copper

Copper is a mineral that, when present in high dietary levels, serves as a growth stimulant for pigs. It is commonly incorporated into their diet in the form of copper sulfate, copper chloride, tribasic copper chloride, and copper citrate, functioning as a growth promoter, the growth performance, and the antioxidant status of weaned pigs [104,105,106]. Copper has antimicrobial properties; the presence of copper causes bacteria to be eliminated quickly because copper ions are more toxic to bacteria due to damage to the bacterial membrane, an increase in reactive oxygen species, and an increase in bacterial DNA degradation [107]. In an experiment conducted by Perez et al. [108], nursery pigs fed Cu-rich sources (250 mg/kg diet) diets with pharmacological levels of ZnO (3000 mg/kg) and antibacterial agents showed a higher growth response. The antimicrobial effectiveness of Cu is associated with its valency [109,110,111]. According to Saphier et al. [109], when compared with divalent, the monovalent Cu is a strong agent against *E. coli*. Similar to Zn, antimicrobial resistance has become a concern in the use of Cu as a growth promoter. Studies have indicated that pharmacological doses of Cu in the diet increase the antimicrobial resistance of *E. coli* in the intestines of pigs [111]. Therefore, these more efficient sources of Cu must be considered in order to reduce its use in swine diets, reducing environmental pollution and the risk of microbial resistance.

### 5.4. Probiotics

Probiotics are live cultures that are included in animal diets to colonize and increase the concentration of the intestinal microflora, thereby competing with the intestinal microflora and preventing the colonization of harmful pathogens [112]. The three main categories of commonly used probiotics are *Bacillus* spp. (spore-forming Gram-positive bacteria), lactic acid-producing bacteria (such *as Lactobacillus*, *Bifidobacterium*, and *Enterococcus*), and yeast [2,73,113,114]. In a previous study by Lewton et al. [113], it was found that including multi-strain *B. subtilis*-based probiotics in the nursery diets has a positive effect on intestinal morphology and improves nursery pig immune function by increasing plasma IgA concentrations by 20% and increasing the expression of the anti-inflammatory cytokine IL-10 in the jejunum. *Bacillus subtilis* supplementation improves the BW (20%) and feed efficiency (13%) of pigs infected with F18^+^ *E. coli* by improving intestinal integrity and decreasing intestinal permeability, according to the findings of Kim et al. [66]. Becker et al. [83] reported that *B. subtilis* attenuated the effects of the F18^+^ *E. coli* challenge by decreasing *E. coli* shedding, resulting in improvements in intestinal integrity and function. According to Duarte et al. [67], a combination of *Bacillus* sp. and xylanase reduced the fecal score and the concentration of IL6 (27%) in the mucosa of nursery pigs challenged with F18^+^ *E. coli*. The reduction in the inflammatory response led to an increase in villus height (23%) and BW (3%). *Bacillus* spp. use a variety of mechanisms to combat ETEC. These mechanisms include eliciting different reactions, modulating host immune responses by regulating the expression of key cytokines involved in initiating and regulating immune responses, influencing tight junction protein expression, and promoting the growth of beneficial microbes. These mechanisms, taken together, help to improve the host’s intestinal health [87,115]. The use of lactic acid-producing bacteria helps restore intestinal balance [112]. Dietary supplementation of multispecies probiotics (including *L. acidophilus, L. casei, B. thermophilum*, and *E. faecium*) in nursery pigs challenged with F18^+^ *E. coli* demonstrated improved on ADG (51%) and ADFI (44%) due to the reduction in digesta pH, reduction of systemic TNF-α (36%), alleviation of intestinal oxidative stress, and enhancement of intestinal morphology [76].

### 5.5. Prebiotics

Prebiotics play a crucial role in selectively promoting the growth and proliferation of potentially beneficial microorganisms within the gastrointestinal tract [116]. According to Gibson et al. [117], prebiotics must be resistant to gastric acidity, hydrolysis by mammalian enzymes, and gastrointestinal absorption; fermented by intestinal microflora; and selectively stimulated growth and activity of intestinal bacteria associated with health and wellbeing; some examples of prebiotics include oligosaccharides, resistant starch, and non-starch polysaccharides. The mechanisms that can help to inhibit the adhesion of pathogens are through the coating of the epithelial surface of the host, the increase in beneficial bacteria, and the regulation of the decrease in adhesion in pathogens [116]. Yu et al. [118] observed that dietary supplementation of manno-oligosaccharides can alleviate diarrhea and alteration of the intestinal epithelium in nursery pigs exposed to ETEC, suggesting that the mechanisms of action are linked to increased tight junction protein expression and distribution, reduced cell apoptosis, and inflammation, and increased antioxidant capacity in the intestinal epithelium.

It is important to note that there are pieces of evidence that high doses of prebiotics can harm nursery pigs [119], especially in the presence of ETEC infections [120]. Prebiotics are substances that promote the growth of beneficial bacteria, which can be advantageous for overall intestinal health. However, when administered in excessive amounts, prebiotics can alter the digesta viscosity and the balance of the intestinal microbiota, potentially leading to excessive inflammation, particularly in animals that are already facing challenges such as ETEC infections. Another mechanism of prebiotics is to stimulate the immune response, increasing animal defenses against pathogens. However, high doses of prebiotics can overstimulate the immune system, deviating energy and nutrients from growth to immune response, consequently reducing growth performance [121,122].

### 5.6. Postbiotics

Postbiotics, a technology containing non-living microorganisms and/or their constituents that provide a positive impact on the health of the host are a strategy that has been used for many years in feed to promote intestinal health [123]. The most common sources of microorganisms used to produce postbiotics are yeast and bacteria. The proposed mechanism of postbiotics is mainly related to cell wall components and the metabolites produced during fermentation that confer similar benefits to health as the live microbial [124]. Components of yeast cell walls have immunoregulatory properties, preventing pathogenic bacteria such as *E. coli* from adhering to the intestinal lining [114]. *Lactobacillus* spp. has characteristics that confer its use as postbiotics. Xu et al. [7], evaluating the effects of *Lactobacillus* fermentate on the intestinal health of nursery pigs challenged with F18^+^ *E. coli,* concluded that the *Lactobacillus* fermentate, as a postbiotic, increased BW (14%), ADFI (20%) and the diversity of beneficial microbiota, reducing jejunal epithelial damages after F18^+^ *E. coli* challenge.

### 5.7. Phytobiotics

Phytobiotics encompass plant-derived, natural bioactive compounds that influence appetite, endogenous secretions, and animal growth and provide a spectrum of health benefits due to their antimicrobial, anti-inflammatory, and antioxidant properties. This term encompasses a range of applications, including essential oils, botanicals, and extracts obtained from various herbs and spices [125]. Phytobiotics have the potential to be used to mitigate the damages caused by *E. coli* infection, considering their antimicrobial, anti-inflammatory, and antioxidant properties [126,127,128,129]. Moita et al. [129] reported that a blend of castor oil and cashew nutshell liquid improved the microbiota composition by increasing the abundance of *Lactobacillus* and *Pseudomonas*, while reducing the abundance of *Helicobacter* and *Campylobacter*. According to Caprarulo et al. [88], the use of a blend of phytobiotics was effective in reducing the effects caused by F18^+^ *E. coli* on the growth performance by increasing G:F (51%) and health of nursery pigs by inhibiting the proliferation of pathogens and increasing the abundance of beneficial bacteria. Chang et al. [85] reported that a mixture of thymol, carvacrol, and bitter citrus extract improved immune responses by reducing TNF-α (23%), IL6 (21%), and intestinal integrity resulting in an increased ADG (49%) in nursery pigs challenged with F18^+^ *E. coli*. Jerez-Bogota et al. [87] reported that garlic in combination with apple pomace or blackcurrant reduced the incidence of PWD caused by F18^+^ *E. coli* by reducing the proliferation of pathogens and increasing the growth of beneficial bacteria and the G:F (49%).

## 6. Conclusions

The spread of F18^+^ *E. coli* targeting nursery pigs worldwide has become a significant concern due to its extensive negative impact on health, mortality, and profitability in pig production. From the review of existing research, the F18^+^ *E. coli* infection triggers intestinal inflammation, resulting in elevated IL6 (60%), IL8 (43%), and TNF-α (28%), increasing oxidative damages, and causing a 14% reduction in villus height. In addition, F18^+^ *E. coli* can disrupt the intestinal microbiota by increasing Proteobacteria population, mostly *Helicobacter* spp. further increasing inflammatory responses. The compromised intestinal health results in a 20% decrease in G:F ratio, a 10% lower ADFI, and a 28% reduction in ADG. Dietary intervention should target the reduction of harmful bacteria in order to reduce inflammatory responses in the small intestine of nursery pigs. Among interventions, zinc glycinate reduces TNF-α (26%) and protein carbonyl (45%), boosting ADG by 39%. Lactic acid bacteria decrease TNF-α (36%), leading to a 51% ADG increase; Bacillus spp. reduces IL6 (27%), contributing to a 12% increase in BW. *Lactobacillus* postbiotic enhances BW (14%) and the diversity of beneficial bacteria. Phytobiotics decrease TNF-α (23%) and IL6 (21%), resulting in improved feed efficiency (37%). Additional strategies, including low crude protein, antibacterial minerals, prebiotics, and organic acids, effectively counter F18^+^ *E. coli*.

Therefore, the control of F18^+^ *E. coli* in pig production requires a multifaceted approach that considers nutrition, intestinal health, and intestinal microbiota to reduce the incidence of PWD and its economic and public health consequences. By implementing effective strategies, the impact of F18^+^ *E. coli* outbreaks can be mitigated to ensure the sustainability of pig production and safeguard public health. Continuous investigations of effective dietary interventions are essential in the ongoing effort to control the spread of F18^+^ *E. coli* in pig farms worldwide.

## Figures and Tables

**Table 1:** Impacts of F18^+^ *Escherichia coli* challenge on health and growth responses in nursery pigs, and the nutritional interventions for mitigating deleterious outcomes.

Interventions	Observation		Reference
	F18^+^ *E. coli* (NC vs. PC)	Treatment (PC vs. Treatment)	
Zinc glycinate	Increased fecal score, enterocyte proliferation (44%), TNF-α (27%), protein carbonyl (67%), and MDA (42%) in jejunum; reduced ADG (23%).	Reduced fecal score, IL8 (39%), TNF-α (26%), MDA (31%), and protein carbonyl (45%); increased ADG (39%)	[68]
*B. subtilis*	Increased frequency of diarrhea and neutrophils (33%) in serum; reduced BW (21%), ADG (35%), ADFI (17.8%), and G:F (24%)	Reduced diarrhea, fecal β-hemolytic coliforms, and neutrophils (16%) in serum; increased BW (20%), and G:F (13%) and expression of tight junction protein in the jejunum	[66]
*Bacillus* sp. + xylanase	Reduced BW (7%), ADG (19%), and G:F (18%) and villus height (18%); increased fecal score, Proteobacteria (37%), IL6 (58%), and MDA (215%) in the jejunal mucosa.	Reduced fecal score, IL6 (27%) in the jejunal mucosa; increased BW (3%) and villus height (23%).	[67]
Lactic acid bacteria ^1^	Increased frequency of diarrhea, TNF-α (27%), and villus height (17%).	Improved ADG (51%) and ADFI (44%); reduced TNF-α (36%).	[7]
*Lactobacillus* postbiotic	Reduced BW (12%), ADG (22%), ADFI (17%), increased IL8 (83%), and abundance of harmful bacteria.	Improved BW (14%), ADFI (20%), and increased diversity and abundance of beneficial bacteria	[7]
Garlic and apple pomace or garlic and blackcurrant	Reduced ADG (4%), G:F (4%), increased inflammation, diarrhea, and the abundance of ETEC and other harmful bacteria in the feces.	Improved G:F (51%), inhibited the proliferation of pathogens, and increased the abundance of beneficial bacteria.	[87]
Capsicum oleoresin, garlic botanical, or turmeric oleoresin	Reduced BW (15%), ADG (46%), G:F (53%), and villus height (18%); increased neutrophils (114%) and TNF-α (36%).	Reduced inflammation and fecal score; reduced neutrophils (43%),	[83]
Thymol and carvacrol or a blend of botanicals ^2^	Reduced BW (9%), ADG (26%), ADFI (5%); increased diarrhea, TNF-α (36%), and IL6 (20%) in serum.	Increased BW (8%), ADG (49%), ADFI (5%), G:F (44%), decreased diarrhea, TNF-α (23%), and IL6 (21%) in serum.	[85]
Blend of botanicals + fatty acids ^3^	Reduced G:F (38%) and increased fecal consistency.	Improved G:F (29%) and fecal consistency.	[88]
Osteopontin	Reduced BW (26%), ADG (56%), and G:F (58%); increased blood helper T-cells, total leukocyte counts, and TNF-α (29%).	Increased TNF-α (141%) and restored microbiota composition.	[89]

^1^ *Lactobacillus acidophilus*, *Lactobacillus casei*, *Bifidobacterium thermophilum*, and *Enterococcus faecium*. ^2^ 10% bitter citrus extract, 20% thymol, and carvacrol, 10% of grape seed and grape marc extract, green tea, and hops, and 60% excipient. ^3^ 3.5 to 6 mg/g of caraway oil, 2.3 to 9.0 mg/g of lemon oil, 1.5% of clove powder, 10% of cinnamon powder, 1.5% nutmeg powder, 5% onion powder, 2% pimento powder, 5% orange peel powder, 12.5% peppermint powder and 12.5% chamomile powder + butyric, caprylic, capric, and lauric acid.

## Data Availability

Not applicable.
